# Epigallocatechin-3-gallate suppressed breast oncogenesis via the miR-27a/Wnt/β-catenin pathway axis

**DOI:** 10.3389/fonc.2026.1835026

**Published:** 2026-06-11

**Authors:** Shrila Banerjee, Abul Kalam Azad Mandal

**Affiliations:** Department of Biotechnology, School of BioSciences and Technology, Vellore Institute of Technology, Vellore, Tamil Nadu, India

**Keywords:** breast cancer, cancer therapeutics, EGCG, microRNA, miR-27a, Wnt/β-catenin pathway

## Abstract

**Introduction:**

Breast cancer is a global health concern with increased chemotherapeutic side effects and escalated chemoresistance, requiring alternative therapies. Epigallocatechin-3-gallate (EGCG), a bioactive green tea polyphenol, has exhibited promising anticancer properties. Our study aimed to investigate the tumour-suppressive effect of EGCG and its mechanism in breast cancer.

**Method:**

Anticancer activities, such as cell viability, migration, cell cycle progression, and apoptosis, were evaluated. To investigate the molecular mechanism, miR-27a-3p expression was compared against EGCG, a miR-27a-3p inhibitor, and a combination of EGCG with miR-27a-3p inhibitor treatments. The influence of the individual miRNA inhibitor and its combination with EGCG on cell proliferation was also evaluated to establish the anticancer activity of EGCG via miR-27a-3p modulation. The effects of the individual miRNA inhibitor, EGCG, and their combination treatments on key Wnt/β-catenin pathway markers were examined to further establish the therapeutic route of EGCG via the miR-27a/Wnt/β-catenin pathway axis.

**Result:**

EGCG notably decreased cell viability and migration, induced apoptosis, and initiated G0/G1 cell cycle arrest. Therapeutic investigation of EGCG on MDA-MB-231 cells indicated its regulatory role on oncogenic miR-27a-3p, which was confirmed when miR-27a-3p expression was significantly lower in the combination treatment of EGCG and the miR-27a-3p inhibitor than in the individual inhibitor treatment in this study. The anticancer potency of the miR-27a-3p inhibitor was also revealed against MDA-MB-231 cells, supporting miRNA therapeutics against cancer. Furthermore, EGCG-modulated miR-27a-3p affected key Wnt/β-catenin pathway markers, causing signal suppression, including suppression of a key Wnt-targeted gene and the G1 phase regulator cyclin-D1, which also indicated its potential against breast cancer.

**Discussion:**

These results signify the therapeutic potential of EGCG and its potential implementation through the miR-27a/Wnt/β-catenin pathway axis, resulting in reduced breast cancer proliferation, metastasis, and relapse.

## 1 Introduction

Cancer is recognised as the second leading cause of death in the world and, according to the latest global cancer statistics, breast cancer accounts for the highest number of cases, with 2,261,419 new cases in 2020 (1). Even with early diagnostic capabilities and available treatment strategies, such as chemotherapy, radiation, surgical removal, and hormonal therapy, the mortality rate is not declining in a significant manner. Cancer metastasis, relapse, and acquired chemoresistance contribute to patient mortality, along with numerous side effects of existing cancer therapies, thereby triggering the need for alternative non-cytotoxic therapies.

Epigallocatechin-3-gallate (EGCG) is a major green tea phytochemical from *the Camellia sinesis* plant, which often exhibits significant bioactivity, including anticancer properties. It has been proven to have antiproliferative, antimigration, antimetastatic, anti-invasion, anti-angiogenic, apoptotic, and cell cycle regulatory abilities against breast cancer by targeting numerous regulatory protein markers, i.e., EGFR (2), PI3K/AKT (3), hTERT (4), STAT3-NFkB (5), and their involved signalling pathways. Although the exact mechanism of action is not clear, EGCG was found to signal within cells and alter protein expression after binding to the 67kDa laminin receptor (6). Multiple studies have demonstrated the selective toxicity of EGCG towards cancers. EGCG showed dose-dependent increased toxicity in MCF-7, MCF-7TEM, and MDA-MB-231, whereas when multiple concentrations of EGCG were tested on normal breast cells (MCF-10A), cytotoxicity was only observed after 72 h of 100 µM EGCG treatment (7). Similarly, it also showed selective toxicity towards breast cancer cells, where 50 µM EGCG treatment did not show any significant cytotoxicity for 7 days in MCF-10A. Still, it showed significantly higher toxicity with increased time in MCF-7 (8). An early study also stated that EGCG did not show any toxicity in normal mammary cells compared to cancerous cells (9).

One of the highly involved and crucial pathways in breast cancer is the Wnt/β-catenin pathway, which is observed to be hyperactive in 50% of all breast cancer cases and is linked to a reduced breast cancer survival rate. It is also linked with the aggressive nature and chemoresistance of triple-negative breast cancer (TNBC) (10). The Wnt/β-catenin pathway plays a primary role in maintaining stemness, epithelial-to-mesenchymal transition (EMT), cell differentiation, proliferation, invasion, and metastasis, which are significantly associated with the breast cancer relapse rate (11). The anticancer role of EGCG in regulating the Wnt/β-catenin pathway has already been established in different cancers, including osteosarcoma, lung cancer, colorectal cancer, and renal cancer (12–15), although the fundamental action mechanism is still undefined.

Studies indicated the antiproliferative effect of EGCG-stabilised HIF-1α in lung cancer cells by stimulating miR-210 expression (16), as microRNAs (miRNAs) are endogenous, short, single-stranded, non-coding RNAs that are capable of regulating cell signals by post-transcriptional modification. Similarly, EGCG is also noted to inhibit oncogenesis, induce apoptosis, and enhance chemosensitivity in multiple cancers, including breast cancer, by regulating miRNAs such as miR-1, miR-16, miR-155, and miR-25 (17–20). A detailed comparative study is given in **Table 1**, which shows that relatively few studies have been conducted on EGCG-modulated miRNAs in cancers, indicating the novelty of this study.

**Table 1:** Studies documenting EGCG–miRNA interactions in cancer regulation.

EGCG–miRNAs	Regulatory role	Targeted biomarker	Effect	Selected cancer	Reference
miR-210	Upregulation	Stabilises HIF-1α	Suppresses tumour growth	Lung cancer	(16)
miR-155	Upregulation	Inhibits GRP78, activates NF-κB, and suppresses MDR1	Increases chemosensitivity	Colorectal cancer	(19)
miR-1	Upregulation	Inhibits c-MET expression	Promotes cell cycle arrest and apoptosis	Osteosarcoma	(18)
miR-16	Upregulation	Suppresses Bcl-2	Promotes cell cycle arrest and apoptosis	Liver Cancer	(17)
miR-25	Downregulation	Activates PARP, pro-caspase-3, and pro-caspase-9	Suppresses tumour growth and promotes apoptosis	Breast cancer	(20)
miR-98-5p	Downregulation	Activates P53	Enhances chemosensitivity	Lung cancer	(21)

**Figure 1:**
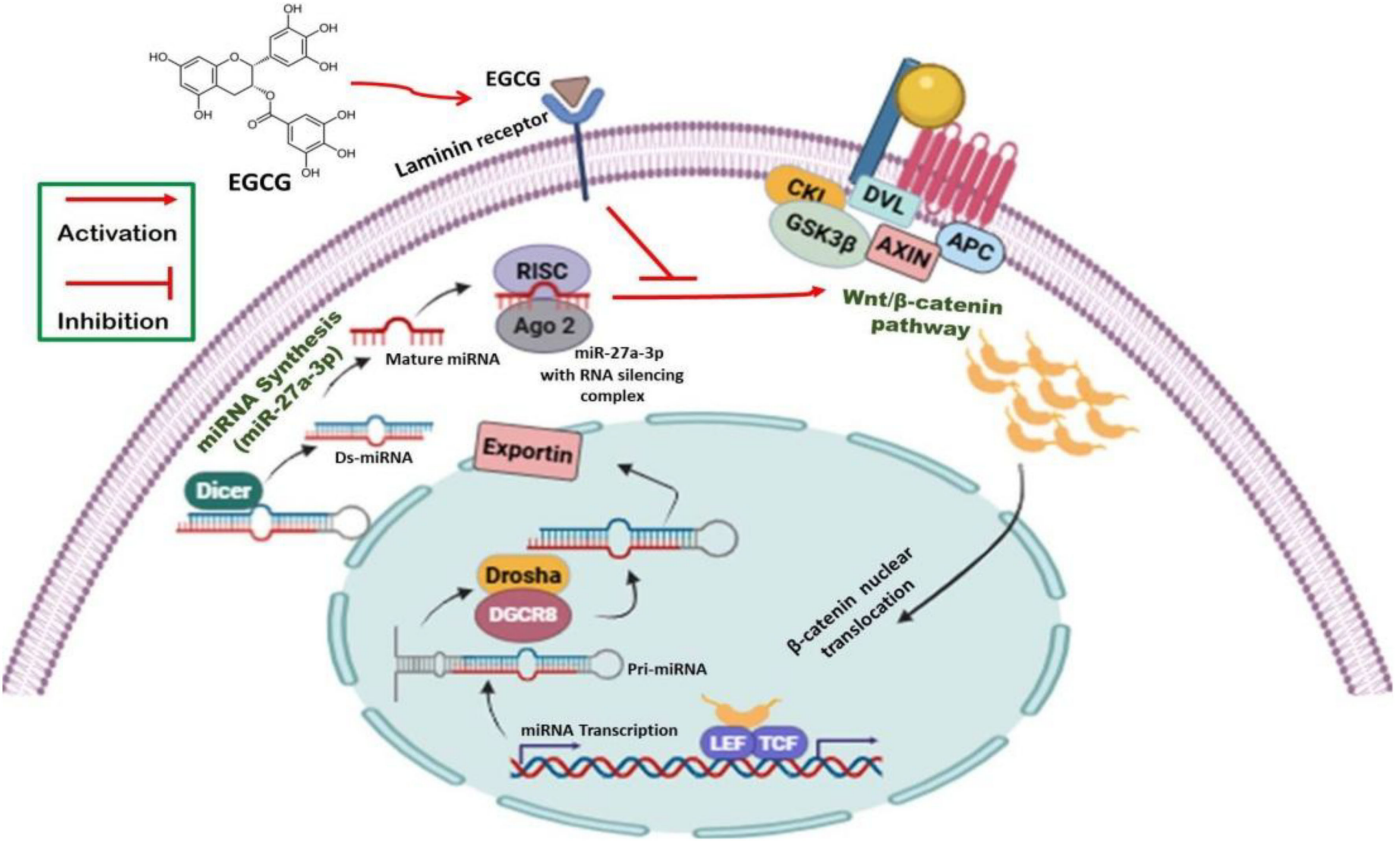
Therapeutic mechanism of EGCG the via miR-27a/Wnt/β-catenin pathway. EGCG inhibits miR-27a-3p expression after binding to the extracellular laminin receptor and exerting a signal within cells. Inhibition of miR-27a-3p expression leads to suppression of the Wnt/β-catenin pathway, which often becomes hyperactive in cancer.

miR-27a, which is often involved with multiple important cancer-related genes, including EGFR, FOXO1, Smad2, MCPH1, and KRAS, in lung, liver, renal, colon, oesophageal, and breast cancers, is a critical miRNA to consider as a prospective target for cancer diagnosis and prognosis (22). While studying the role of miR-27a in breast carcinogenesis, it displayed a significant correlation with breast cancer stage, grade, and immune-molecular type (23). It is known to encourage tumour progression (24) and cell migration (25), enhance chemoresistance (26), and promote differentiation of cancer stem cells (CSCs) (27) in breast cancer by targeting various protein biomarkers, including AKT, GSK-3β, and FBXW7. Moreover, experimental analysis revealed a significant Wnt/β-catenin pathway modulatory role of miR-27a-3p by controlling GSK-3β in TNBCs (28), and our previous study showed differentially expressed miR-27a-3p in response to EGCG on MDA-MB-231 TNBC cells (29), which encouraged further examination of the anticancer mechanistic route of EGCG against breast cancer. Here, we analysed the functional mechanistic role of EGCG against breast cancer, where it exhibited cellular signal regulation via the miR-27a/Wnt/β-catenin pathway axis. This study also indicated the effectiveness of a miR-27a-3p inhibitor as an anticancer treatment against breast cancer cells. **Figure 1** presents a pictorial representation of the hypothetical therapeutic mechanism of EGCG revealed in this study.

## 2 Materials and methods

### 2.1 Material

Sigma Chemical Co., USA, was the supplier of EGCG. Dulbecco’s modified eagle medium (DMEM) with high glucose, antibiotic-antimycotic solution, foetal bovine serum (FBS), acridine orange/ethidium bromide (AO/EB) stain, and MTT [3-(4,5-dimethylthiazol-2-yl)-2,5 diphenyl tetrazolium bromide] stain were all commercially obtained from Himedia Laboratories Pvt. Ltd., India. The transfection reagent and miRCURY LNA miR-27a-3p inhibitor were acquired from Qiagen Sciences, Germany. LEF-1, SOX-4, AXIN-2, and GSK-3-β primers were commercially obtained from Thermo Fisher Scientific Inc., USA. Gsk-3-β, Sox-4, β-actin, and β-catenin antibodies were purchased from Elabscience Biotechnology, Inc., USA. The National Centre for Cell Science, Pune, India, supplied the MDA-MB-231 breast cancer cell line.

### 2.2 Cell culture and miRNA inhibitor transfection

MDA-MB-231 cells were obtained from the National Centre for Cell Science, Pune, India, and cultured using high-glucose-containing Dulbecco’s Modified Eagle Medium (DMEM) mixed with 1x antibiotic-antimycotic solution and 10% foetal bovine serum (FBS) at 37 °C in a 95% humidified atmosphere and 5% CO2. Different concentrations of EGCG were made by dissolving it in the complete culture medium. A working solution of 50 μM miR-27a-3p inhibitor was prepared with the transfection reagent following the manufacturer’s instructions (Qiagen Sciences, Germany) in serum-free medium for miRNA inhibitor transfection.

### 2.3 Cell viability assay

Seeded MDA-MB-231 cells were used to evaluate the antiproliferative effects of EGCG by treatment with three different concentrations of EGCG for 24 h, including the IC50 concentration, which we identified in our previous study (50, 83, and 150 μM). For each well, 10 μl of MTT [3-(4, 5-dimethylthiazol- 2-yl)-2, 5-diphenyltetrazolium bromide] was added the following day, and the cells were incubated at 37 °C for 4 h. The colour formations from dimethylsulfoxide (DMSO)-dissolved formazan crystals after adding to each well were read at 570 nm using a microplate reader and represented in a graphical format.

### 2.4 Cell cycle progression analysis

For cell cycle analysis, seeded cells were given 0, 50, 83, and 150 μM of EGCG for 24 h before collection. Cells were fixed by dropwise addition of 80% ice-cold ethanol to the cell pellet and incubated at 4 °C. Soon before analysis, cells were centrifuged, and 500 μL PBS containing 0.04 mg/mL propidium iodide (PI) and 0.1 mg/mL RNase was added to the cell pellets. The pellets were incubated in the dark for 30 min at 37°C before cell cycle analysis using a CytoFLEX Flow Cytometer (Beckmen Coutler, California, USA).

### 2.5 Protein level analysis

For further validation, expression differences of two G1 phase-regulatory proteins, Cyclin-D1 and E1, were evaluated following treatment with three selected EGCG concentrations (50, 83, and 150 μM). Protein levels of β-catenin, Sox-4, and Gsk-3-β after treatment with 83 µM EGCG, 50 μM miR-27a-3p inhibitor (as per standard kit protocol), and a combination of EGCG with the miR-27a-3p inhibitor were evaluated. Cells were seeded in 60 mm plates and grown to 90% confluency. Cell extracts were prepared from MDA-MB-231 cells after the selected treatments for 48 h, and western blotting was performed. Protein concentration was determined using Lowry’s method (30). Approximately 100 μg of protein samples was taken to perform SDS-PAGE (12%) using a polyvinylidene fluoride (PVDF) membrane (GE Healthcare, Chicago, USA). After transfer, the membrane was incubated with a blocking buffer (5% BSA in Tris-buffered saline with 0.1% Tween 20) and probed with appropriate primary (Cyclin-D1, Cyclin-E1, β-catenin, Sox-4, Gsk-3-β, and β-actin as a reference control) and secondary antibodies (Elabscience, USA). The experiments were performed in triplicate, and all blots were developed using a chemiluminescent reagent and scanned using a gel documentation system. Optical densities of the bands were quantified using ImageJ.

### 2.6 Wound-healing assay

To check the anti-migratory effect of EGCG, MDA-MB-231 cells were seeded and grown to 90% confluency in a 6-well plate. Vertical wounds were made in each well using a sterile pipette after overnight starvation in serum-free medium. EGCG treatment (0, 50, 83, and 150 μM) was given the next day. Cell migration rates were monitored and photographed under a Labomed TCM 400 inverted microscope (Labo America, California, USA) at four different time intervals (0, 6, 12, and 24 h) by calculating and comparing wound widths.

### 2.7 Cell death assay

To check the apoptotic ability of EGCG, cells were seeded and grown in a 6-well plate. EGCG treatment (0, 50, 83, and 150 μM) was given for 24 h, and an AO/EB (acridine orange/ethidium bromide) (Himedia, India) double-staining assay was used for apoptosis detection. A total of 100 μL of AO/EB from a mixture of 100 µg/mL AO and 100 µg/mL EB in PBS was added to each well of the 6-well plate and observed under an Olympus CX41 upright fluorescence microscope (Olympus, Tokyo, Japan).

### 2.8 Real-time quantitative PCR

To determine the regulatory function of EGCG on miR-27a-3p expression, seeded MDA-MB-231 cells were exposed to six treatments over the course of 24 h: 50, 83, and 150 µM EGCG, negative inhibitor, miR-27a-3p inhibitor, and a combination of EGCG and the miR-27a-3p inhibitor. Differences in miR-27a-3p expression following the selected treatments were compared with the untreated sample. After converting the total RNAs to cDNA using the miRNeasy Mini Kit (Qiagen Sciences, Germany), qRT-PCR analysis was carried out using the MiRCURY LNA miRNA PCR starter kit (Qiagen Sciences, Germany) and the MiRCURY miR-27a-3p PCR assay (YP00206038), with U6 serving as the reference control (YP02119464). The CFX96 Touch Real-Time PCR Detection System, manufactured by Bio-Rad, USA, was used for real-time detection, and the ΔΔCT method was used to examine the variations in expression.

Expression validation of the Wnt/β-catenin pathway was performed by comparing untreated cells with six treatments over the course of 24 h, namely 50, 83, and 150 µM EGCG, negative inhibitor, miR-27a-3p inhibitor, and a combination of EGCG and the miR-27a-3p inhibitor using four key components, namely, LEF-1, SOX-4, AXIN-2, and GSK-3-β. Cells were seeded and grown in 60 mm plates before receiving 0, 50, 83, and 150 µM EGCG treatment for 24 h. The cells were washed with PBS, and total RNAs were extracted by the Trizol reagent (Takara, Japan) method. Extracted RNAs were converted to cDNA using the RT reagent kit (Takara, Japan) according to the manufacturer’s instructions. For qRT-PCR, primers were designed using the primer-BLAST tool from NCBI, and the following primer pairs were used: forward and reverse primers of *GAPDH* (5’TCAAGGCTGAGAACGGGAAG3’, 5’CTAGTTGCCTCCCCAAAGCA3’), *LEF-1* (5’AGGAACATCCCCACACTGAC3’, 5’AGGTCTTTTTGGCTCCTGCT3’), *SOX-4* (5’CCACACCATGAAGGCGTTC3’, 5’CCGAGCTGGTGCAAGACC3’), *AXIN-2* (5’CTGGCTTTGGTGAACTGTTG3’, 5’AGTTGCTCACAGCCAAGAGA3’), and *GSK-3-β* (5’TCAAGGCTGAGAACGGGAAG3’, 5’CTAGTTGCCTCCCCAAAGCA3’). *GAPDH* was used as an internal reference gene. The qRT-PCR was carried out with the QuantiNova SYBR green PCR kit (Qiagen Sciences, Germany) and gene primers from Thermo Fisher Scientific Inc., USA. Expression levels were analysed using the ΔΔCt method.

### 2.9 Statistical analysis

Statistical analysis was performed with a t-test. The experimental data were presented as mean ± SD. The results were considered significant when p < 0.005, p < 0.01, or p < 0.05.

## 3 Result

### 3.1 Strong cytotoxic effect of EGCG against breast cancer cells

**Figure 2:**
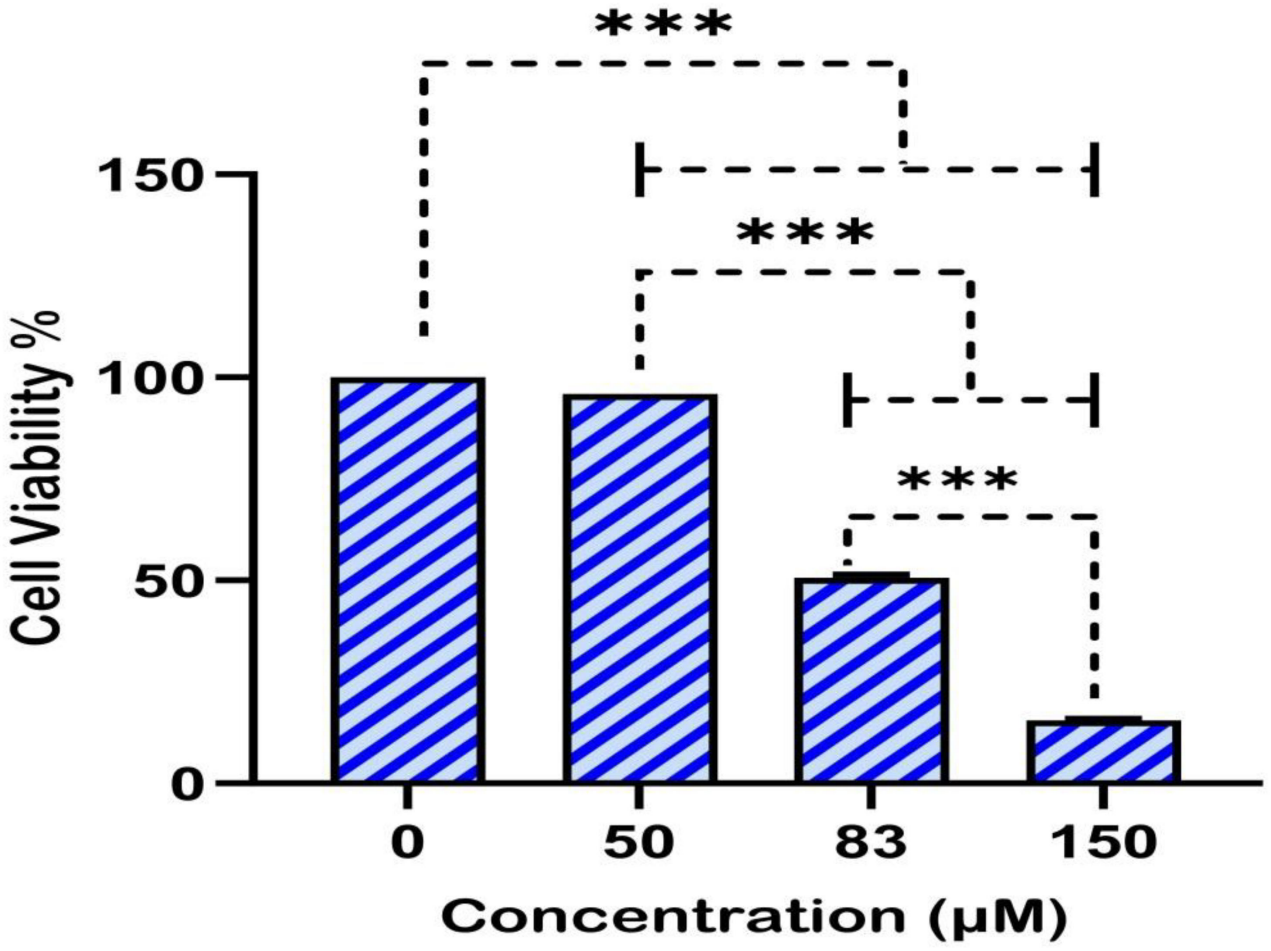
The effect of different concentrations of EGCG (50, 83, and 150 µM) on the viability of MDA-MB-231 cells. A dose-dependent reduction in cell viability was noticed. The significance of differences between the control and all the treatments is indicated by “*”. Significance levels of p < 0.05 (“*”), p < 0.01 (“**”), or p < 0.005 (“***”) are denoted. Data are presented as mean ± SD.

After treatment, a visible reduction in cell viability was noticed through colorimetric evaluation. Decreased cell viability was observed with increasing EGCG doses. MDA-MB-231 cells showed higher viability when treated with a lower concentration of EGCG. Approximately 70% of cells were viable after 50 μM EGCG, which reduced to 50% of cells at 83 μM EGCG treatment. After 150 μM EGCG treatment, only 37% of cells were observed to be viable. These data confirm the potent cytotoxicity of EGCG against breast cancer cells (**Figure 2**).

### 3.2 EGCG induced G0/G1 phase arrest in MDA-MB-231 cells

**Figure 3:**
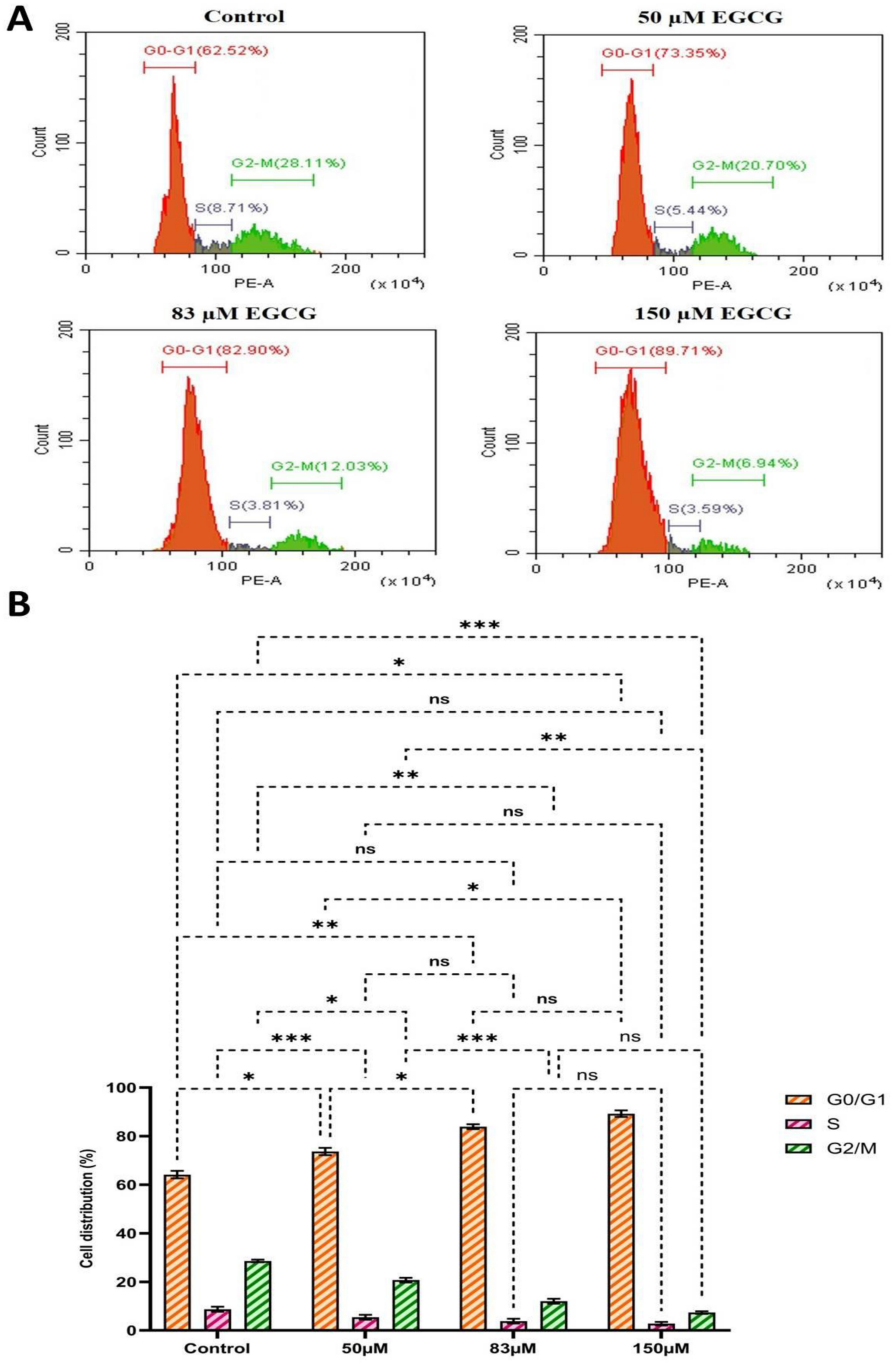
G0/G1 cell cycle arrest in breast cancer cells by EGCG in a dose-dependent manner. **(A)** Cell cycle analysis of MDA-MB-231 cells treated with 0, 50, 83, and 150 μM concentrations of EGCG. **(B)** Graphical representation of quantitative cell cycle analysis of MDA-MB-231 cells treated with 0, 50, 83, and 150 μM concentrations of EGCG, showing induced G0/G1 phase arrest. The significance of differences between the control and all the treatments is indicated by “*”. Significance levels of p < 0.05 (“*”), p < 0.01 (“**”), or p < 0.005 (“***”) are denoted. Data are presented as mean ± SD.

A dose-dependent increase in cell numbers was observed in the G0/G1 phase after EGCG treatment. Treatment with 50, 83, and 150 μM EGCG resulted in 73.35%, 82.90%, and 89.71% of cells in the G0/G1 phase, respectively, whereas in untreated cells (control), it was 62.52%. Simultaneously, the dose-dependent increase in the percentage of cells in the G0/G1 phase led to a reduction in the percentage of cells in the G2/M phase, with 28.11% of untreated cells in the G2/M phase, whereas 50, 83, and 150 μM EGCG-treated cells showed 20.70%, 12.03%, and 6.94%, respectively. Our results indicated an increased cell cycle inhibitory capability of EGCG at the G0/G1 phase in MDA-MB-231 breast cancer cells (**Figures 3A, B**).

### 3.3 Validation of G0/G1 arrest induction by EGCG through cyclin expressions

**Figure 4:**
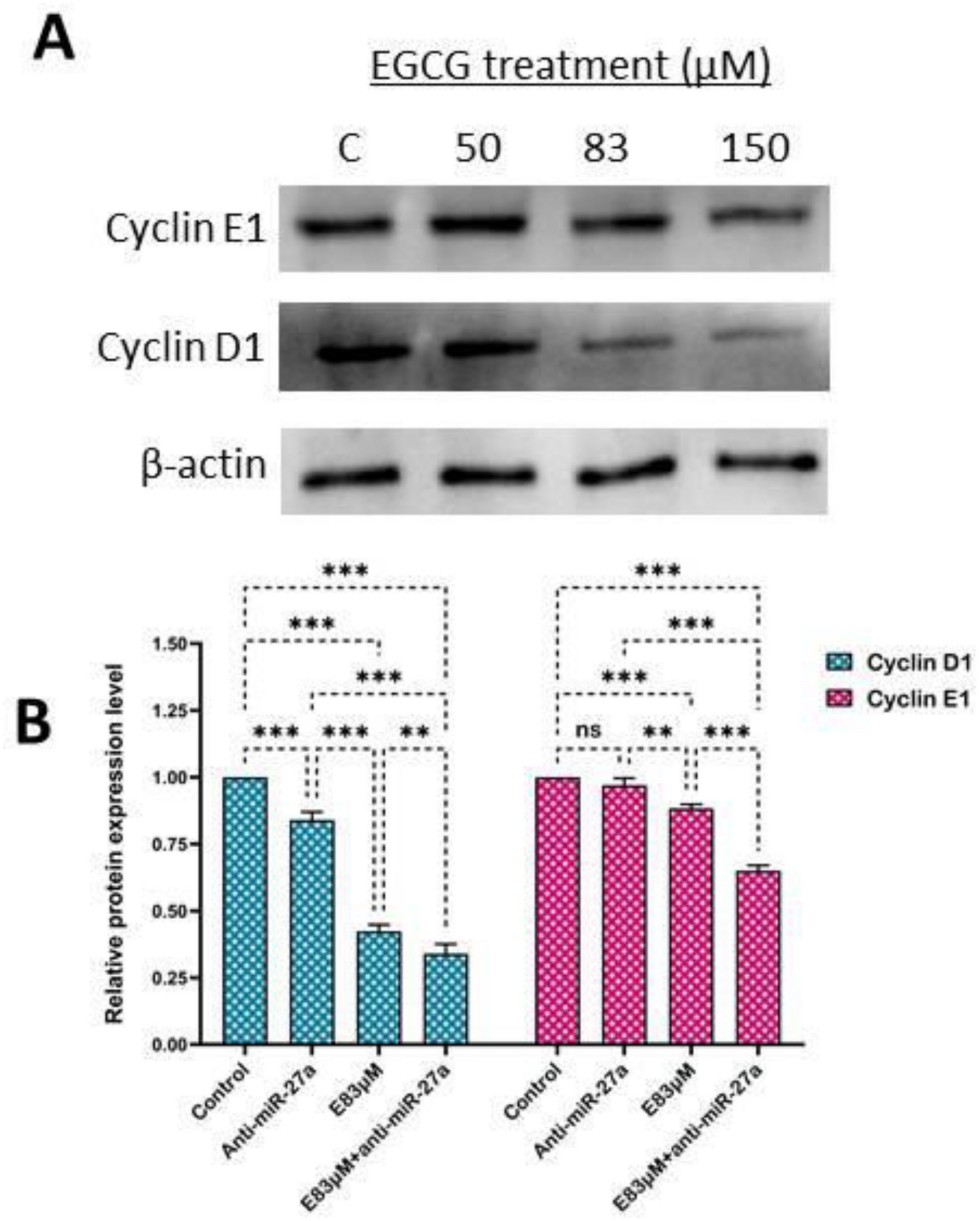
Validation of G0/G1 cell cycle arrest in breast cancer cells by EGCG in a dose-dependent manner. **(A)** Cell cycle validation of MDA-MB-231 cells treated with 0, 50, 83, and 150 μM concentrations of EGCG by western blot. **(B)** Graphical representation of quantitative Cyclin-D1 and Cyclin-E1 expression in MDA-MB-231 cells treated with 0, 50, 83, and 150 μM concentrations of EGCG. The significance of differences between the control and all the treatments is indicated by “*”. Significance levels of p < 0.05 (“*”), p < 0.01 (“**”), or p < 0.005 (“***”) are denoted. Data are presented as mean ± SD.

To further validate the cell cycle inhibitory efficacy of EGCG, the expression of the G1 phase regulatory proteins Cyclin-D1 and E1 was checked. EGCG treatments (50, 83, and 150 μM) significantly decreased the expression of these proteins compared to untreated cells, which indicated EGCG-induced cell cycle arrest of MDA-MB-231 cells at the G0/G1 phase. Protein expression differences were calculated using the housekeeping protein β-actin as a reference. Dose-dependent decreases in the expression of Cyclin-D1 (0.84-, 0.42-, and 0.35-fold change) and Cyclin-E1 (1-, 0.90-, and 0.65-fold change) were observed. Pictorial and graphical representations of differential Cyclin-D1 and Cyclin-E1 expression are given in **Figures 4A, B**.

### 3.4 EGCG visibly caused apoptosis in MDA-MB-231 cells

**Figure 5:**
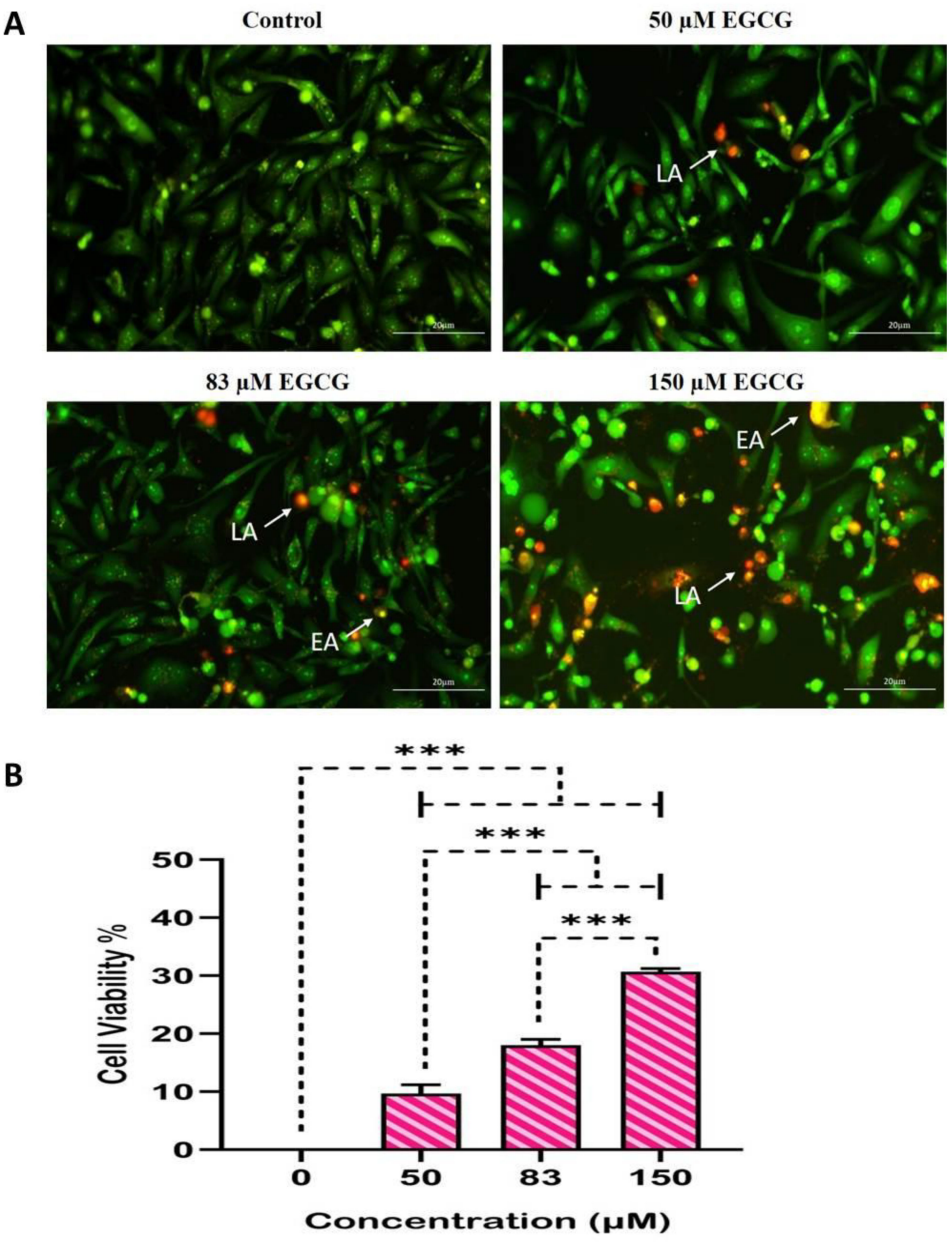
EGCG influences breast cancer cell apoptosis ability in a dose-dependent manner. **(A)** AO/EB staining of MDA-MB-231 control, 50, 83, and 150 μM concentrations of EGCG-treated cells; Green: living cell; EA: early apoptosis (yellowish orange); LA:;ate apoptosis (reddish orange). **(B)** Graphical representation of quantitative apoptotic analysis after different doses of EGCG. The significance of differences between the control and all the treatments is indicated by “*”. Significance levels of p < 0.05 (“*”), p < 0.01 (“**”), or p < 0.005 (“***”) are denoted. Data are presented as mean ± SD.

The apoptotic ability of EGCG was investigated using nuclei and cells stained with a fluorescent AO/EB mixture, where live and apoptotic cells were distinguished through green and orange coloration. No orange cells were observed in the untreated cancer cells, nor was there any significant variation in the cell nuclei or cell membrane integrity. EGCG-treated samples showed evidence of apoptotic cells with significant chromatin condensation, fragmented nuclei, and cell membrane disruption. The yellow-stained cells were early apoptotic, and the orange-stained cells were late apoptotic. Even though the treatment exposure time was short, a dose-dependent increase in apoptotic cells was observed (**Figure 5**). A distinct number of apoptotic cells confirmed the apoptotic ability of EGCG against MDA-MB-231 cells.

### 3.5 EGCG inhibited MDA-MB-231 cell migration

**Figure 6:**
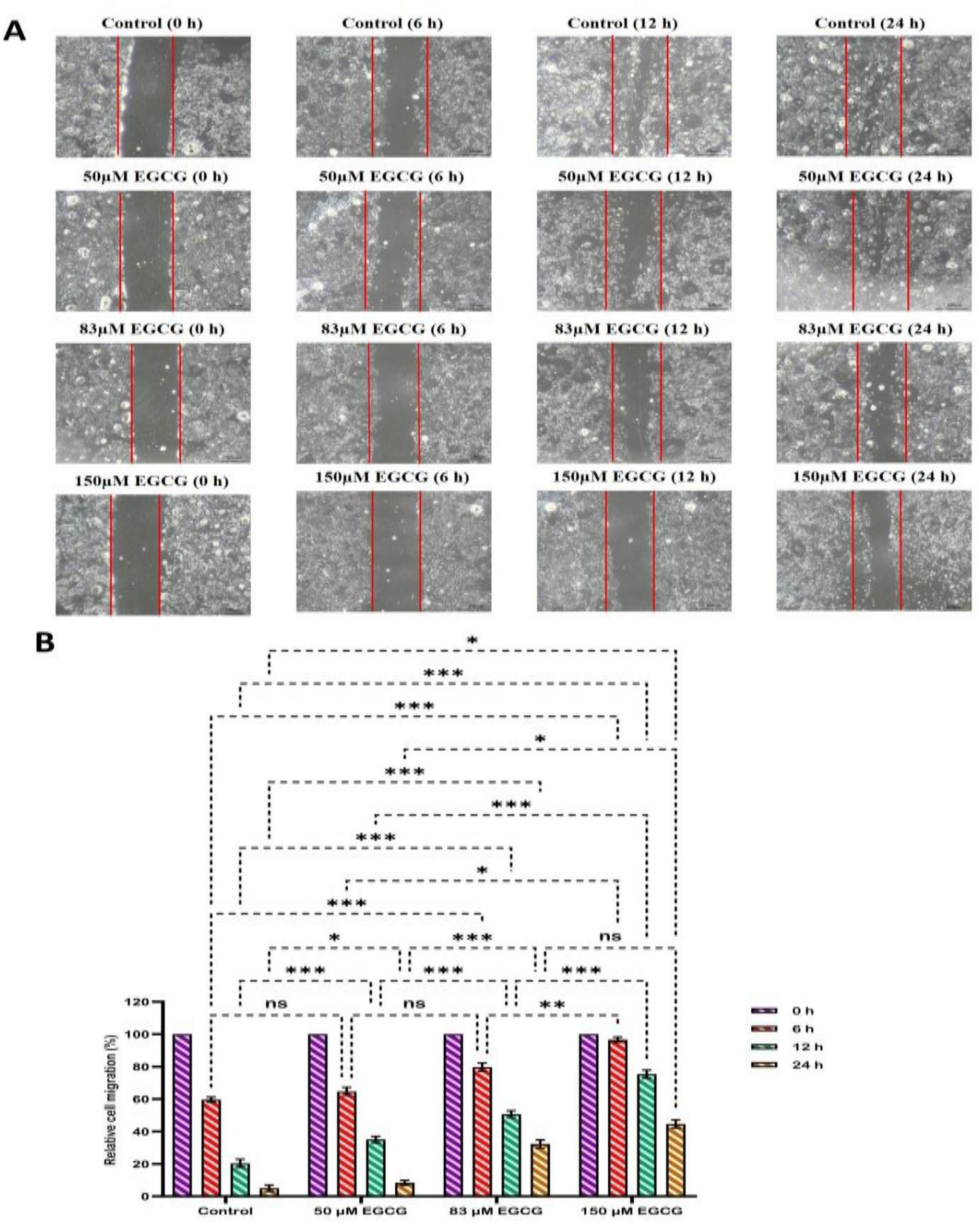
EGCG reduced breast cancer cell migration ability in a dose-dependent manner. **(A)** Wound-healing assay of MDA-MB-231 control, 50, 83, and 150 μM concentrations of EGCG-treated cells for the observation of cell migration rate at different time intervals (0 h, 6 h, 12 h, and 24 h). **(B)** EGCG’s antimigration properties were evaluated quantitatively after 6, 12, and 24 h of incubation. The significance of differences between the control and all the treatments is indicated by “*”. Significance levels of p< 0.05(“*”), p <0.01(“**”), or p< 0.005 (“***”) are denoted. Data represented as mean ± SD.

Another attribute of cancer is the ability of cells to migrate and maintain cell-cell interactions by forming a two-dimensional monolayer. A wound-healing assay was performed to evaluate the anti-migratory efficacy of EGCG against MDA-MB-231 cells by observing the gap reduction rate resulting from cell migration after creating a scratch on the cell monolayer. Higher gap reduction was observed in untreated cells compared to EGCG-treated cells. The cell migration rate was observed to be inversely proportional to the EGCG treatment concentration. The migration rate of MDA-MB-231 cells at 50 μM EGCG after 6 h, 12 h, and 24 h of incubation was 25%, 55%, and 95%, respectively, compared to 0 h. The migration rate decreased with increased treatment concentration and treatment time interval (6, 12, and 24 h) (**Figures 6A, B**). The cell migration rate of MDA-MB-231 cells treated with 83 μM EGCG was 18%, 45%, and 70%, respectively, whereas 150 μM EGCG-treated MDA-MB-231 cells showed 5%, 15%, and 65% cell migration (**Figures 6A, B**). These data further demonstrate the anticancer efficacy of EGCG against breast cancer.

### 3.6 miR-27a-3p expression was downregulated by EGCG

**Figure 7:**
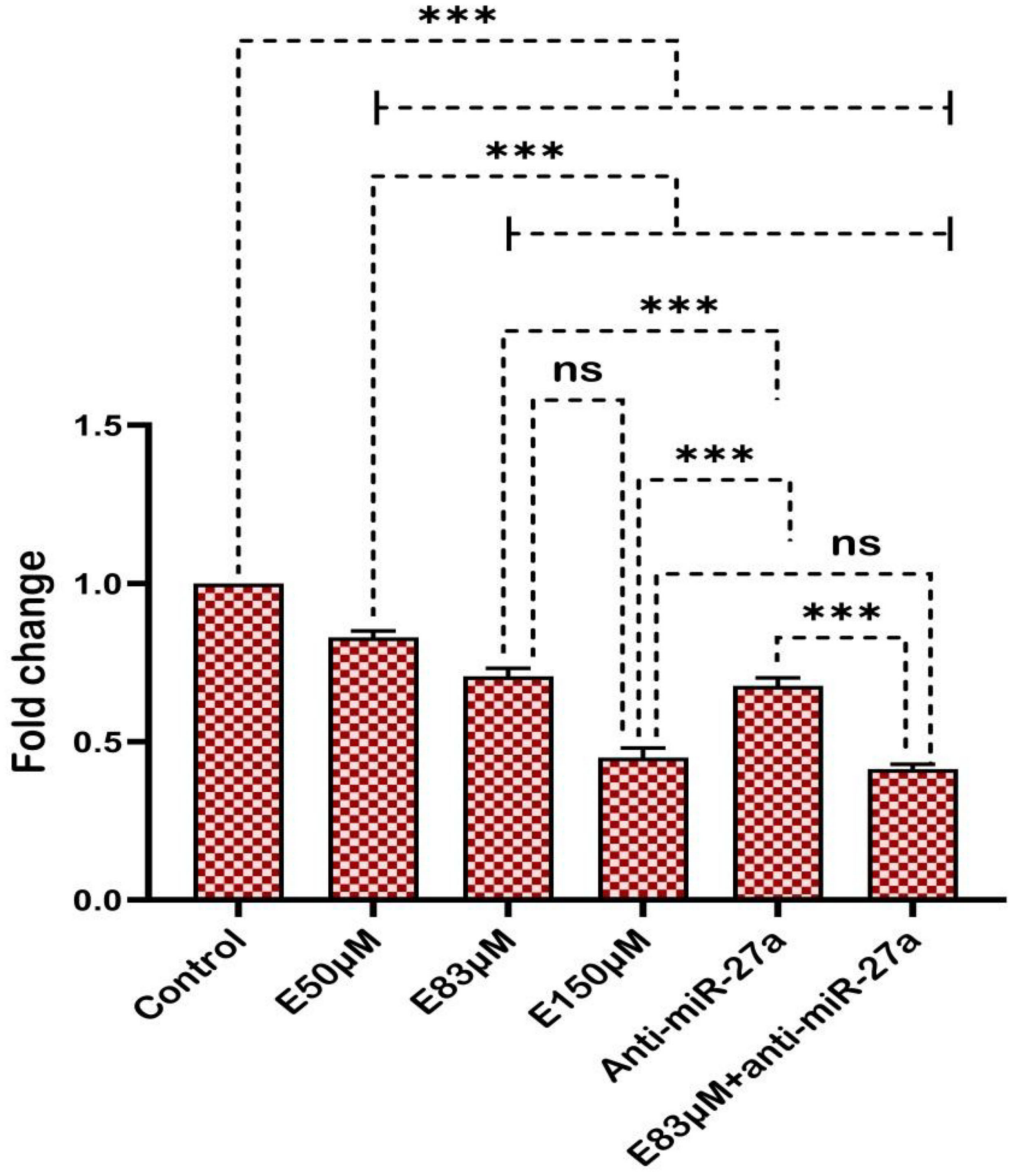
The real-time PCR validation of miR-27a-3p downregulation by EGCG. Expression variation of miR-27a-3p was observed with increasing doses of EGCG. A significantly decreased expression of miR-27a-3p after combination treatment with EGCG and the miR-27a-3p inhibitor, compared to individual treatments, indicates miR-27a-3p influential ability of EGCG. The significance of differences between the control and all the treatments is indicated by “*”. Significance levels of p < 0.05 (“*”), p < 0.01 (“**”), or p < 0.005 (“***”) are denoted. Data are presented as mean ± SD.

A gradual decrease in miR-27a-3p expression was noticed with increasing EGCG concentration. miR-27a-3p expression suppression was also noticed after transfection with the inhibitor, which indicated the positive uptake of the miR-27a-3p inhibitor. Furthermore, the expression of the miRNA decreased further after combined treatment with the miR-27a-3p inhibitor and 83 μM EGCG compared to individual inhibitor treatment, which validated the miR-27a-3p modulatory efficacy of EGCG and suggested a synergistic effect. Log2fold expression differences of 0.82, 0.71, 0.45, 0.67, and 0.40 for hsa-miR-27a-3p were noticed after 50, 83, and 150 μM EGCG, miR-27a-3p inhibitor, and combined miR-27a-3p inhibitor and EGCG treatments, respectively, relative to the untreated control (**Figure 7**). These data demonstrate the miR-27a-3p modulatory efficacy of EGCG.

### 3.7 Anti-proliferation of EGCG through miR-27a-3p suppression

**Figure 8:**
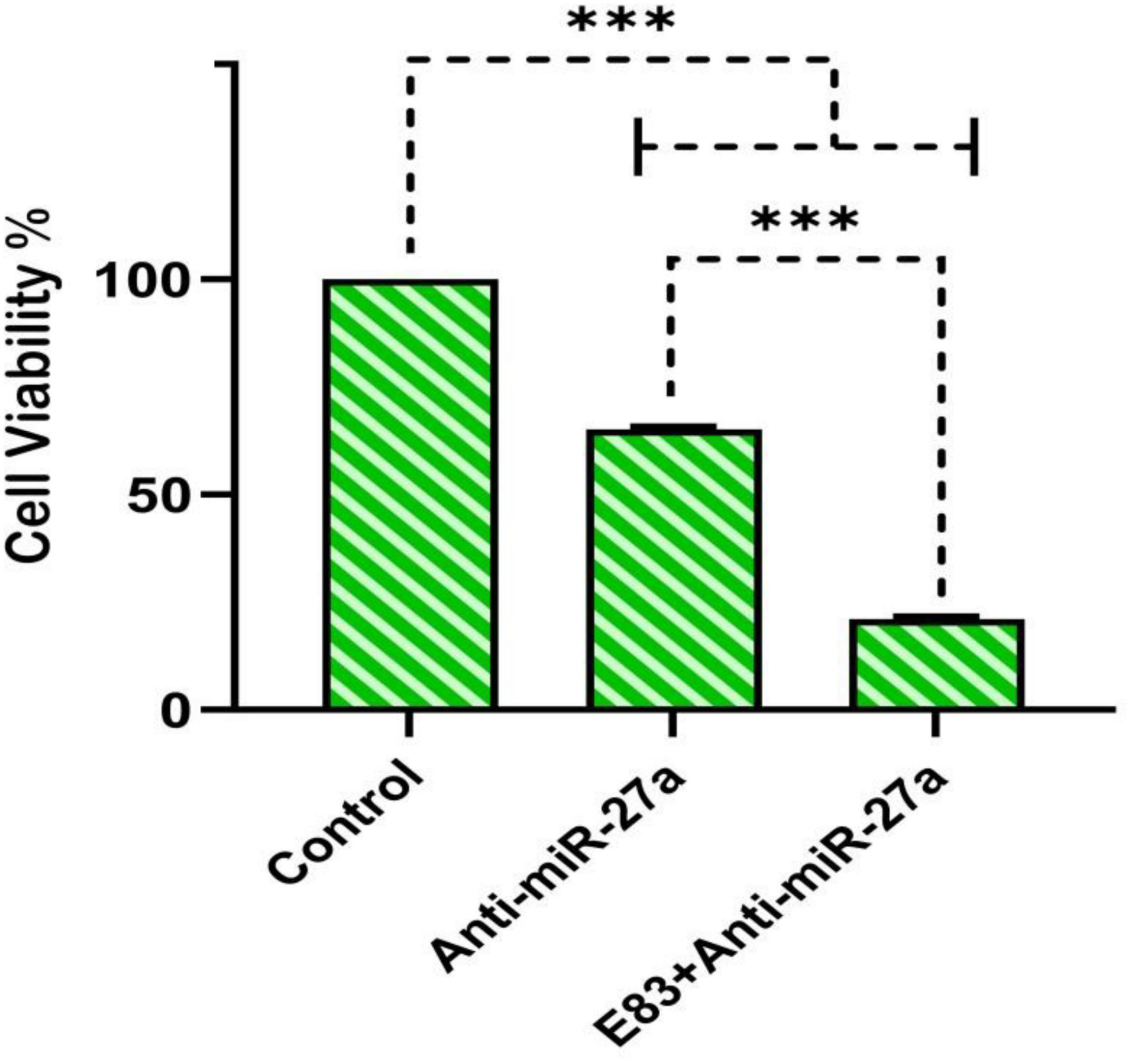
Antiproliferation of EGCG through miR-27a-3p suppression validated by MTT assay. Significantly decreased viability was noticed in the combination of the miR-27a-3p inhibitor with EGCG compared to individual treatment, indicating the miR-27a-3p influential ability of EGCG. The significance of differences between the control and all the treatments are indicated by “*”. Significance levels of p < 0.05 (“*”), p < 0.01 (“**”), or p < 0.005 (“***”) are denoted. Data are presented as mean ± SD.

Decreased cell viability was noticed after treatments. Approximately 61% of MDA-MB-231 cells were viable after treatment with the individual miR-27a-3p inhibitor treatment, whereas only 30% of cells were viable after combined treatment with EGCG with the miR-27a-3p inhibitor (**Figure 8**). This result reveals the anticancer potential of the miR-27a-3p inhibitor against MDA-MB-231 breast cancer cells and the synergistic anticancer potency of the combined treatment with EGCG and the miR-27a-3p inhibitor. The antiproliferative effect of the miR-27a-3p inhibitor was significantly higher than that of 50 μM EGCG treatment on breast cancer cells. The result confirmed mimicking the anticancer effect of the miR-27a-3p inhibitor compared to EGCG and a synergistic anticancer effect when combined with EGCG.

### 3.8 EGCG and miR-27a-3p inhibitor regulated the Wnt/β-catenin pathway

**Figure 9:**
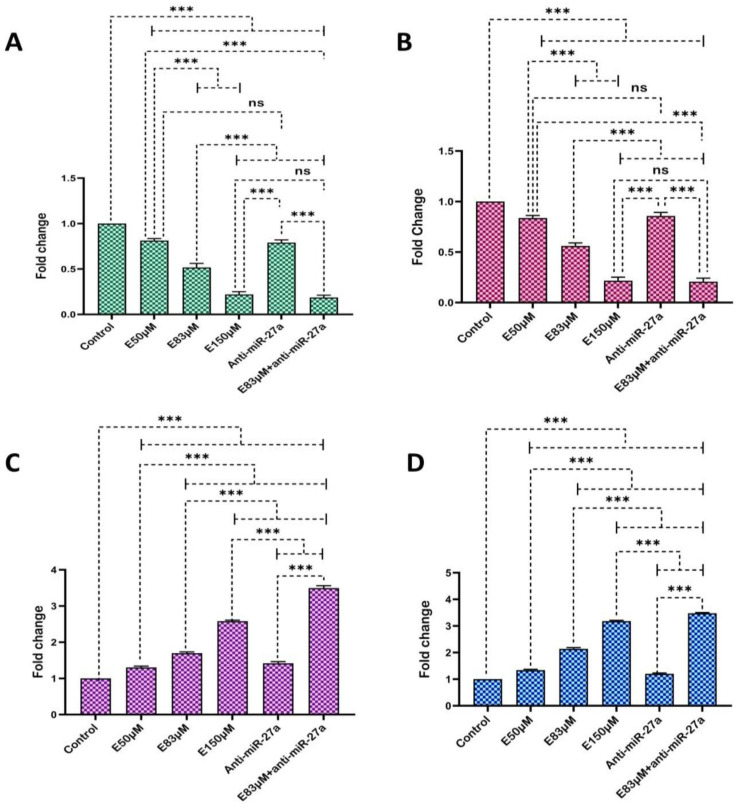
Validation of Wnt/β-catenin pathway regulation of EGCG via miR-27a modulation against MDA-MB-231 cells by qRT-PCR. Differential expression of each marker was observed following treatment with EGCG, miR-27a inhibitor, and their combination. **(A)** Log2 fold change of LEF-1; **(B)** Log2 fold change of SOX-4; **(C)** Log2 fold change of AXIN-2; and **(D)** Log2fold change of GSK-3-β. MDA-MB-231 cells were treated with 0, 50, 83, and 150 μM EGCG, miR-27a-3p inhibitor, and a combination of 83 μM EGCG with the miR-27a-3p inhibitor. The significance of differences between the control and all the treatments is indicated by “*”. Significance levels of p < 0.05 (“*”), p < 0.01 (“**”), or p < 0.005 (“***”) are denoted. Data are presented as mean ± SD.

**Figure 10:**
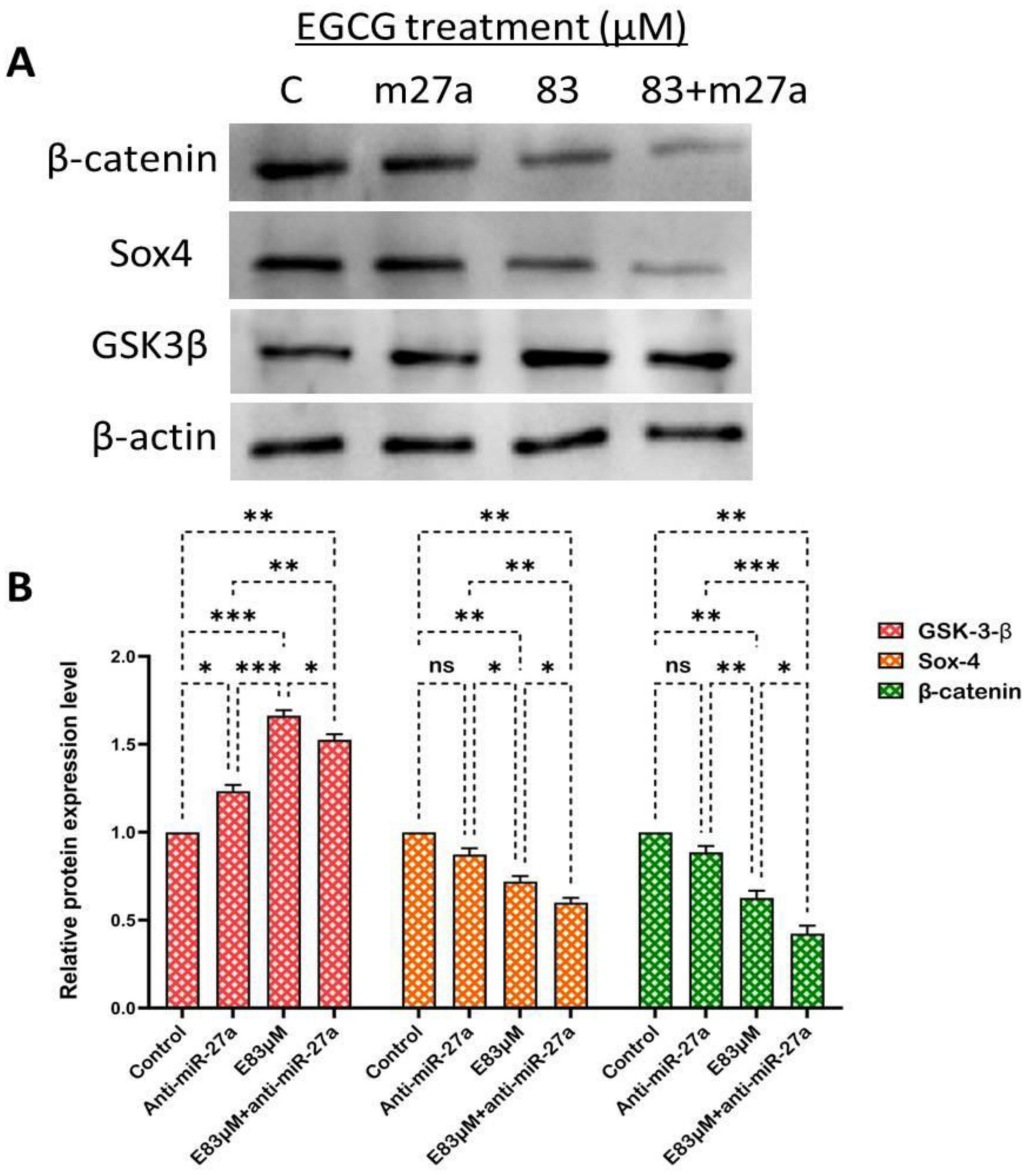
Validation of Wnt/β-catenin pathway regulation of EGCG via miR-27a modulation against MDA-MB-231 cells by western blot. **(A)** Protein expression of Gsk-3β, Sox4, and β-catenin on western blot. **(B)** Graphical representation of quantitative expression of Gsk-3β, Sox4, and β-catenin. MDA-MB-231 cells were treated with 0, 83 μM EGCG, miR-27a-3p inhibitor, and a combination of 83 μM EGCG with the miR-27a-3p inhibitor. The significance of differences between the control and all the treatments is indicated by “*”. Significance levels of p < 0.05 (“*”), p < 0.01 (“**”), or p < 0.005 (“***”) are denoted. Data are presented as mean ± SD.

Changes in gene expression levels amongst the untreated, 50, 83, and 150 µM EGCG-treated, miR-27a-3p inhibitor-treated, and combined miR-27a-3p inhibitor and EGCG-treated cells were calculated using the -ΔΔct method, keeping *GAPDH* as the reference. *LEF-1* showed fold changes of 0.81, 0.52, 0.22, 0.78, and 0.18, whereas *SOX-4* showed fold changes of 0.84, 0.55, 0.22, 0.85, and 0.21 downregulation in control vs 50, 83, and 150 µM EGCG-treated, miR-27a-3p inhibitor-treated, and combined miR-27a-3p inhibitor and EGCG-treated MDA-MB-231 cells, respectively. In contrast, *AXIN-2* showed fold changes of 1.30, 1.70, 2.58, 1.42, and 2.70, whereas *GSK-3-β* showed fold changes of 1.34, 2.15, 3.18, 1.20, and 3.48 upregulation in control vs 50, 83, and 150 µM EGCG-treated MDA-MB-231 cells, respectively. Results showed significantly decreased expression of LEF-1 and SOX-4, whereas expression of AXIN-2 and GSK-3-β increased in response to different doses of EGCG. Treatment with the miR-27a-3p inhibitor also showed similar expression changes in LEF-1, SOX-4, AXIN-2, and GSK-3-β, mimicking the therapeutic effect of EGCG. Combined treatment of the miR-27a-3p inhibitor with EGCG showed a greater differential expression pattern of LEF-1, SOX-4, AXIN-2, and GSK-3-β, which confirms the involvement of EGCG and miR-27a in the modulation of the Wnt/β-catenin pathway (**Figure 9**). EGCG showed prominent anticancer effect against breast cancer cells via the miR-27a/Wnt/β-catenin pathway axis. EGCG showed a prominent anticancer effect against breast cancer cells via the miR-27a/Wnt/β-catenin pathway axis. A similar expression pattern was also noticed in the protein level study, where GSK-3-β and sox-4 showed similar expression changes. The main component of the targeted pathway, i.e., β-catenin, also showed decreased expression due to the treatment with the miR-27a inhibitor and EGCG (**Figure 10**).

## 4 Discussion

Breast cancer has always been the most common cancer in women, and recently it became the most diagnosed cancer (1). Even though the occurrence rate of breast cancer is the highest, the mortality rate is promisingly low, approximately 6.9% (1), and disease relapse is correlated with the death rate (31). Thus, numerous studies are being conducted on new and combination therapies to control breast cancer growth and disease relapse. Here, we demonstrated the anticancer potency of EGCG against MDA-MB-231 TNBC cells and examined the molecular mechanism of its therapeutic role. EGCG evidently decreased miR-27a-3p expression, which also correlated with suppressed proliferation and migration, increased cell cycle arrest, and apoptosis. EGCG and the inhibited miR-27a-3p also inhibited the hyperactive Wnt/β-catenin pathway, which also correlated with decreased expression of the Wnt-target gene cyclin-D1, signifying G0/G1 phase arrest. Silencing of miR-27a-3p also affected the expression of key Wnt/β-catenin pathway markers. Collectively, these observations demonstrated that EGCG treatment effectively suppressed breast oncogenesis through the miR-27a-3p/Wnt/β-catenin pathway axis.

Multiple studies have indicated the anticancer potency of the non-cytotoxic, antioxidant-rich green tea catechin EGCG, especially against breast cancer (32). It also exhibited promising chemosensitisation results against paclitaxel- and doxorubicin-resistant breast cancer cells (33, 34). EGCG exhibits potent anticancer therapeutic ability, chemopreventive ability, and the ability to reduce breast cancer recurrence (35). In the majority of anticancer studies, EGCG arrested the cell cycle at G0/G1 (36–42); similarly, in our study, EGCG caused G0/G1 phase arrest in MDA-MB-231 TNBC cells (**Figures 2A, B**), although it exhibited G2/M phase arrest in the ER+ breast cancer cell line MCF-7 (20). Furthermore, in our western blot study, significant dose-dependent decreases in the expression of Cyclin-D1 and Cyclin-E1 were observed, which validated the flow-cytometric analysis (**Figures 3A, B**). This may suggest that hormonal receptor expression influences the mechanistic pathway of EGCG action. The involvement of p53 and Bcl2 was noticed in the ER+ MCF7 breast cancer cell line, causing significant apoptosis and decreased cell proliferation (43). However, the therapeutic mechanism of EGCG against breast cancer is yet to be discovered.

EGCG has promisingly shown multiple other anticancer properties, including antimigration, apoptosis, and antiproliferation against multiple cancers. A 48-h treatment with 74.71 µg/mL EGCG showed distinct apoptosis in liver cancer (44), whereas nano-encapsulated EGCG also induced prominent apoptosis by disrupting mitochondrial activity in a cervical cancer cell line (45). Similarly, 24 h of EGCG treatment in MDA-MB-231 cells also induced apoptosis, although fewer apoptotic cells were observed after AO/EB staining, which may indicate that a longer treatment exposure time is required for a highly significant effect (**Figure 4**). EGCG also showed dose-dependent anti-migratory ability in this study (**Figures 5A, B**), a feature that contributes significantly to the aggressiveness of cancer, influencing its metastatic nature. Even though breast cancer has a higher survival rate, its mortality rate is found to be uniquely related to cancer relapse and its migration ability (46).

For a deeper understanding, the regulatory mechanisms of EGCG at the genomics level were investigated, as our previous study clearly indicated the miRNA modulatory efficacy of EGCG in MDA-MB-231 breast cancer cells, showing anticancer activity. Gene expressions are often modulated at the post-transcriptional phase by endogenous, non-coding, single-stranded miRNAs, thereby controlling multiple cellular pathways. In our previous study, one of the highly targeted pathways was the Wnt/β-catenin pathway, which also supported its significant cancer-promoting impact in breast oncogenesis (29, 47). Multiple miRNAs are known to modulate this cell proliferative pathway, e.g., miR-203, miR-1, and miR-493, which are capable of inhibiting FZD receptors, whilst miR-142, miR-34, miR-155, and miR-26 are capable of targeting the β-catenin destruction complex. miR-200a, miR-141, and miR-340 are capable of targeting β-catenin directly, whilst miR-30c, miR-612, and miR-139 often target the transcription region of the Wnt/β-catenin pathway controlling oncogenesis (48).

One of the highly involved miRNAs from our last study was miR-27a-3p, which indicated promising anti-breast cancer therapeutic potential of EGCG via miRNA modulation. Although fewer studies have been conducted on the regulatory role of EGCG on miR-27a, especially in oncogenesis, EGCG has been shown to influence miR-27a-3p, causing a reduction of lipid accumulation and sensitisation to insulin resistance (49, 50). In this study, miR-27a-3p expression was modulated following different doses of EGCG treatment, miR-27a-3p inhibitor treatment, and combined miR-27a-3p and EGCG treatment, which confirmed the regulatory efficacy of EGCG against miR-27a-3p (**Figure 6**). Higher antiproliferative efficacy was also noticed after combined miR-27a-3p inhibitor and EGCG treatment compared to individual miR-27a-3p treatment, which also confirmed the anticancer activity of EGCG through miR-27a-3p modulation (**Figure 7**). The same miRNA has been shown to regulate the Wnt/β-catenin pathway by targeting RXRα, FOXO1, SFRP1, and GSK3β in colorectal, ovarian, gastric, and breast cancers, respectively (28, 51–53). In this study, key Wnt/β-catenin pathway markers (LEF-1, SOX-4, AXIN-2, and GSK-3-β) showed differential expression after different doses of EGCG compared to untreated MDA-MB-231 cells, which indicated a dose-dependent pathway-modulatory effect. On the contrary, expression differences of the same markers were noticed after miR-27a-3p inhibitor treatment, which indicated the Wnt/β-catenin pathway-targeting role of miR-27a-3p. Moreover, the expression difference increased after the combined miR-27a-3p inhibitor and EGCG treatment, which further indicates the Wnt/β-catenin pathway-modulatory ability of EGCG via miR-27a-3p.

## Data Availability

The original contributions presented in the study are included in the article/supplementary material. Further inquiries can be directed to the corresponding author.
